# Permeability evolution of Bentheim Sandstone at simulated georeservoir conditions

**DOI:** 10.1038/s41598-023-42826-3

**Published:** 2023-09-27

**Authors:** Marco Fazio, Michael R. Chandler, Martin Sauter

**Affiliations:** 1 Department of Applied Geology, University of Göttingen, Goldschmidtstrasse 3, 37077 Göttingen, Germany; 2 School of Geosciences, University of Edinburgh, Edinburgh, UK; 3 Leibniz-Institute of Applied Geophysics, Hannover, Germany

**Keywords:** Geology, Hydrogeology, Geothermal energy

## Abstract

Bentheim Sandstone is considered a suitable conventional georeservoir rock even at great depth because of its mineral composition, homogeneity, micro- and macrostructure, and is also used as a reference material in rock deformation tests. However, a full characterization of the permeability at representative depths has never been performed. Here we report new experimental data where the permeability of Bentheim Sandstone is measured both with a simultaneous variation and with a sequential variation of three different variables to simulate georeservoir conditions. The results indicate a decrease in permeability with simulated increasing depth until 2–3 km, followed by a partial permeability recovery until 4–5 km depth. During the exhumation path, initially, permeability is unaffected, but at shallow depths, a sharp increase in permeability is observed, likely due to microcracking. These variations are a consequence of a complex interaction between stress, pore pressure and temperature, highlighting the importance of experiments considering all three variables when studying the evolution of permeability at depth. These results will aid with the accurate estimation of permeability at different georeservoir conditions.

## Introduction

Bentheim Sandstone (BS) is a sedimentary rock formed during the Lower Cretaceous in a shallow marine environment in the western part of the Lower Saxony Basin. It takes its name after the German city Bad Bentheim, at the border with the Netherlands. Its main outcrop forms a 9-km-long east–west ridge centered on Bad Bentheim^1^. Because of its well-sorted grain size, lateral continuity and homogeneity at the block scale (no bedding planes are visible in hand specimens), BS is considered a reference material in rock deformation experiments. In addition, BS is almost entirely composed of quartz crystals, and, because of its well-sorted grain size and a well-connected equant pore space^2^, is both highly porous and highly permeable making it a perfect reservoir rock to study rock-fluid interactions and transport processes even a great depths^3–6^.

In fact, BS is a suitable deep, warm aquifer for potential low-cost geothermal energy in the Netherlands^7^, a case study rock for anhydrite cementation in georeservoirs^8^ and one of the most important aquifers in the North German Basin^9^. The top of the BS formation is at a depth of about 1.5 km, but BS can be found at more than 2 km depth and has been buried as deep as 3.5 km^10^, so it is crucial to understand its properties at different depths.

While earlier studies focused mostly on geological and paleontological aspects of the BS, more recent ones, particularly in the last two decades, have experimentally investigated the physical, hydraulic, mechanical and chemical properties^5,11,12^. Permeability, in particular, is one of the most important rock properties of porous materials and its indirect determination is quite difficult to achieve. In fact, while a relationship between porosity and permeability has been noted, a model applicable to all rocks does not exist. Still, it is rather specific for the investigated rocks under specific conditions^13,14^. Therefore, direct measurements of permeability and its evolution at different in-situ conditions are essential to characterize a georeservoir.

With increasing depth, an inverse relationship between lithostatic pressure and permeability has been observed in many rocks, both sedimentary and igneous^6,14,15^. Nevertheless, the permeability of BS is unaffected by the increase of effective pressure^2,6^ (${\sigma }_{eff}=\sigma -{p}_{p}$). In these experiments, the pore pressure (*p*_*p*_) was kept constant so that the increase in effective pressure represented the increase in lithostatic pressure.

This low-pressure sensitivity is due to the low structural anisotropy and compositional variation of BS, with no bedding layers visible to the naked eye, negligible permeability anisotropy affecting the fluid flow, and a well-connected network of equant (low aspect ratio) pores with little presence of microcracks (high aspect ratio). High aspect ratio cracks tend to close more easily than low aspect ratio pores at increasing hydrostatic pressure, therefore the latter impedes permeability reduction. In fact, over the investigated range of 5–90 MPa, the porosity of BS samples decreases by only 4%, compared to the 21% porosity reduction found in Crab Orchard sandstones (COS, less porous and permeable, with a mixture of cracks and pores). Over the same pressure range, COS experiences a permeability reduction of more than 90%, in perfect agreement with the inverse relationship between lithostatic pressure and permeability^2^.

A slight dependence of BS permeability on effective pressure has been observed in two recent studies. Benson et al.^13^ noted a small permeability decrease with increasing effective pressure, associated with the decrease of fracture density. Although the equant-pores provide the largest contribution to the bulk permeability, the slight permeability reduction at higher pressure is mainly caused by a change in microcrack permeability. Dautriat et al.^16^ also discovered a slight, non-linear change in BS permeability with applied pressure, with the parallel-to-bedding permeability decreasing more than the perpendicular-to-bedding permeability. In addition, Dautriat et al.^16^ demonstrated that end effects at the interface between the sample and the loading piston led to localized compaction damage at the sample surface, which caused significant permeability reduction. However, by measuring radial permeability and so bypassing the end surfaces, the measurement of permeability is not affected by end effects.

In the brittle regime, an increase in differential stress (${\sigma }_{diff}={\sigma }_{1}-{\sigma }_{3}$) typically leads to an initial compaction phase and permeability reduction, followed by a dilation phase and ultimately by sample failure, both causing permeability enhancement^14^. However, Dautriat et al.^16^ observed no permeability increase during the dilation phase. They did report a minor permeability increase at sample failure, but permeability remained lower than at initial conditions. Overall, permeability decreased once the differential stress had been reduced to its initial value, implying that the permeability reduction due to compaction was greater than the increase during failure. This decrease, mostly perpendicular to the sample’s axis, has been associated with the formation of the shear band, followed by sliding and grain crushing along it. Although the fault gouge may not significantly affect the permeability of the whole sample^17^, the permeability measured across a damage zone is highly reduced^16,18^.

The role of strain in both plastic and ductile regimes has been investigated by Vajdova et al.^19^, where BS samples underwent significant axial strains up to 14%. Once failure or stress plateau occurred, BS either experienced strain-softening at low effective pressure or strain-hardening at high effective pressure with the formation of shear localization and compaction localization respectively. Similarly, to the case of differential stress, permeability continues to decrease by around one order of magnitude after the formation of a fracture as more grain crushing is induced on the sample at elevated axial strain. In addition, when the sample does not fail, the formation of compaction bands in the plastic regime also impedes the fluid flow through the sample, causing permeability reduction by up to 3 orders of magnitude. This proves that even thin bands of low permeability can cause a considerable decrease in bulk permeability when the flow intersects the low permeability band at a high angle.

The mineral assembly also contributes to the evolution of permeability. In fact, the presence of clay minerals reduces the permeability by clogging pore spaces when dislodged from their original positions at a high flow rate, even with low clay concentrations as in BS^20^.

With increasing depth, effective pressure, differential stress and lithostatic pressure are not the only variables that vary. Pore pressure change has a contrasting effect on permeability compared to a change in effective pressure: while the latter tends to close pores and cracks, the former tends to open these. An increase in temperature may either induce thermal microcracking (leading to higher permeability), thermal expansion of the mineral matrix or dissolution and recrystallization reactions (both leading to lower permeability). Pressure conditions, rock microstructure and mineral assembly determine which mechanism eventually dominates^14,21^. In addition, rocks may undergo a complex stress history, with cycles of loading and unloading, which affects rock properties. Despite being a common benchmark material in geomechanics, the effects of all of these variables on permeability have yet to be investigated in BS, even while having been investigated on various other rocks^21–24^.

Therefore, the objective of this study aims at closing the above-mentioned gaps, by performing laboratory experiments and studying both individually and collectively the variables affecting the permeability of Bentheim sandstone at increasing depths.

## Materials, equipment and method

Cubic blocks (300 × 300 × 300 mm) of BS were collected from the Romberg quarry in Gildehaus, ca. 4 km west of Bad Bentheim (Germany). Bedding layers were not visible at the block scale nor in the hand specimen, but their orientations could be determined by ultrasonic velocity measurements. Cylindrical samples were cored, cut and ground to reach an approximate diameter of 100 mm and length of 250 mm. Samples were manufactured with the axis both parallel and perpendicular to the bedding layers.

**Figure 1:**
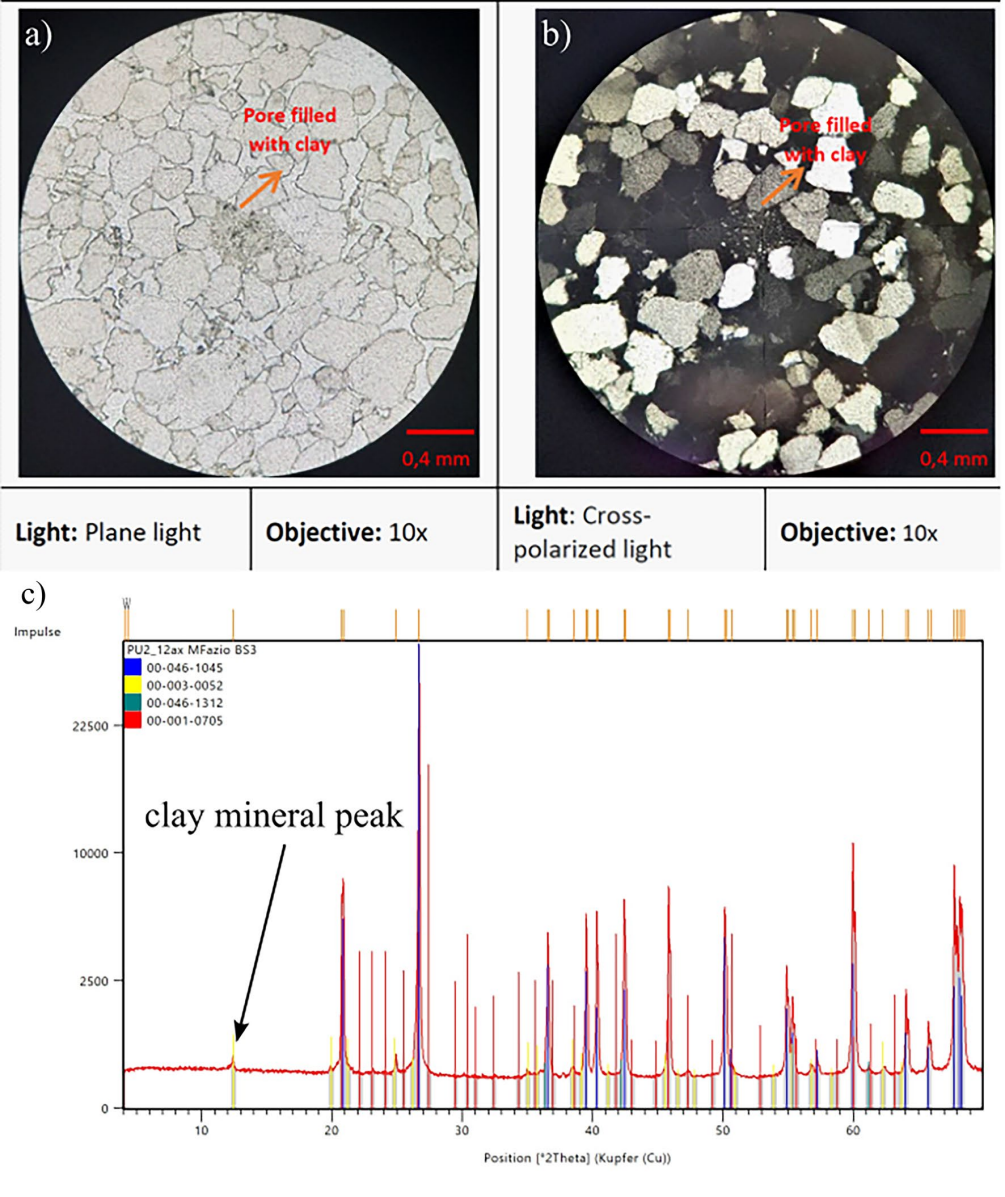
(**a**) plane light and (**b**) cross-polarized light photos of Bentheim sandstone in thin sections. The well-sorted nature of the BS is apparent, as well as the ubiquitous presence of quartz crystals. A pore-filling clay mineral is also shown (at the flat end of the arrow). (**c**) X-ray diffraction analysis on BS, showing the clay mineral peak. The main, blue-colored peaks are related to quartz, while the others are associated with feldspars and oxides.

Thin section and XRD analyses (Fig. 1) confirm the dominant presence of quartz grains, as well as fewer feldspars, oxides and clay minerals within the pore space (Fig. 1a). The pore space is well interconnected (average pore interconnectivity > 68%) and consists of mostly low-aspect ratio pores with longest dimension equal to 0.125–0.5 mm. Sub-angular, fine- to medium-sand size grains characterize the solid part of BS, with no visible fractures or deformation structures.

Porosity (*n*) and density (*ρ*) of BS were determined by water saturation and either caliper (for regular cores) or the buoyancy technique (for irregular specimens)^25^, yielding *n* = 24.37 ± 0.05% and *ρ* = 1987 ± 13 kg/m^3^. By measuring the P-wave velocities (*v*_*p*_) at room temperature/pressure conditions it was possible to determine the orientation of the bedding layers, yielding a *v*_*p*_ of 2540 ± 104 and 2248 ± 97 m/s parallel and perpendicular to bedding respectively.

**Figure 2:**
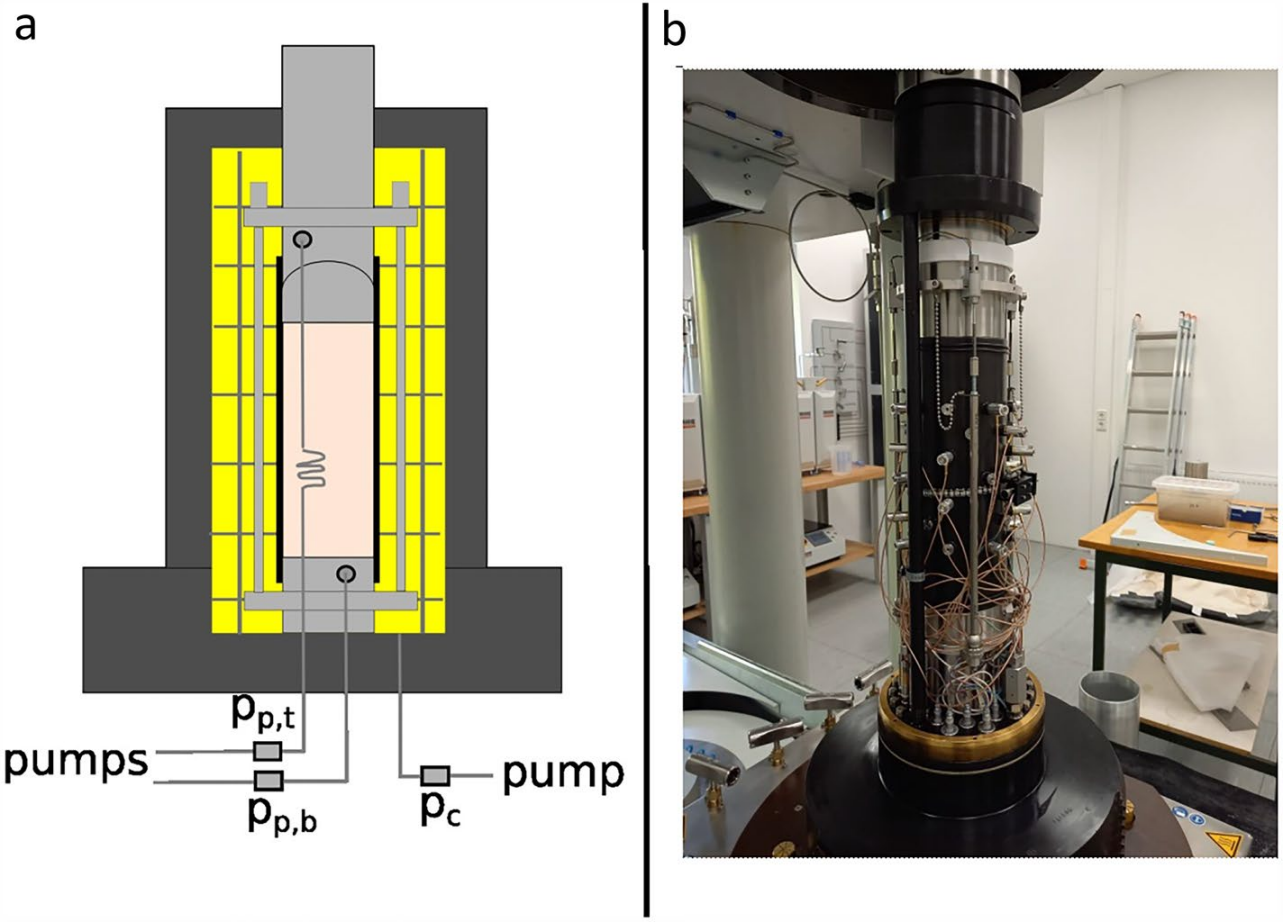
(**a**) schematic of the sample assembly and the internally heated pressure cell; (**b**) photo of the sample assembly with the AE transducers (grey cylindrical elements) embedded in the engineered Nitrile jacket (black). The axial and radial LVDTs are also visible in the photo.

Permeability experiments were performed in an internally heated, servo-controlled triaxial apparatus (Fig. 2a) at the Laboratory of Experimental Hydro-Geomechanics (LEHG) at the University of Göttingen. The apparatus is capable of simulating pressure/temperature conditions of depths of up to ≈ 6 km (*σ*_*1*_ = 200 MPa, *σ*_*3*_ = 100 MPa, *p*_*p*_ = 100 MPa, *T* = 180 °C), with a 2 × 180 mL double piston pump providing upstream and downstream pore pressure. In addition to pressure transducers and temperature thermocouples, the apparatus is equipped with 3 LVDTs recording the axial displacement and one LVDT recording radial displacement. These data were recorded at a sampling frequency of 1 Hz.

The sample cored with the axis perpendicular to the bedding planes was placed in a simple Nitrile jacket. The samples cored with the axis parallel to the bedding layers were placed in an engineered Nitrile jacket^26^, fitted with ports to host an array of 16 piezoelectric transducers (Fig. 2b) to monitor Acoustic Emissions (AEs) as a result of microcracking, and perform ultrasonic surveys to study the evolution of P-wave velocities of the sample as the experimental conditions changed. These transducers have an excitation frequency range of 500 to 1000 kHz, and data were recorded at a sampling frequency of 10 MHz.

Because of the high porosity of Bentheim sandstone, each sample was saturated with water before the experiment and then placed into the jacket, and subsequently into the confining cell, so to avoid the use of a large volume of water pumped just to saturate the sample. The sample was positioned inside the cell, which was filled with the confining medium (a high-flash-point oil). After this step, the initial conditions were established (see lower values for each variable in Table 1). Permeability was then firstly measured at the initial conditions, and then at each step of pressure/temperature conditions, by imposing a pore pressure difference (Δ*p*_*p*_) = 1 MPa between the upstream and downstream pumps, which resulted in a Δ*p*_*p*_ ≈ 20 kPa in the cell because of the high permeability of the sandstone. For the same reason, the steady-state-flow method and Darcy’s Law (Eq. 1) were applied, in which the permeability was measured when, for a constant Δ*p*_*p*_, a constant and equal in value (but opposite in sign) flow rate was measured at both ends of the sample^23^.

**Table 1:** Experimental stress, pore-pressure and temperature conditions of the permeability tests on BS.

Sample	Axis orientation to the bedding layers	σ_3_ (MPa)	p_p_ (MPa)	*σ*_*eff*_ (MPa)	σ_1_ (MPa)	*σ*_*diff*_ (MPa)	T (°C)	AE array
BS8	Perpendicular	2–100	1–50	1–50	2.4–120	0.4–20	18–165	no
BS13	Parallel	2–80	1–40	1–40	2.4–96	0.4–16	17–134	yes
BS16	Parallel	3–80	2–70	1–78	5–82	2	18–140	yes

*σ*_*3*_: horizontal stress; *p*_*p*_: pore pressure; *σ*_*eff*_: effective pressure; *σ*_*1*_: vertical stress; *σ*_*diff*_: differential stress; *T*: temperature; AE: Acoustic Emission.

1 $$Q= \frac{k*{\Delta p}_{p}*A}{\mu *L}$$

*Q* = volumetric flow rate (m^3^/s).

*k* = permeability (m^2^).

*Δp*_*p*_ = pore pressure difference (Pa).

*A* = sample cross-sectional area (m^2^).

*µ* = viscosity (Pa*s).

*L* = sample length (m).

Considering that the samples are 250 mm long and 100 mm wide and that the changes in sample length and diameter are negligible (less than 1% in length), *A* and *L* can be assumed constant. The water viscosity (*µ*) at different pressure and temperature conditions was derived from^27^. The pore pressure difference (*Δp*_*p*_) was measured by pore pressure transducers placed near the cell (Fig. 2a) while the volumetric flow rate (*Q*) was measured at both the upstream and downstream pressure pumps.

For each change in *σ*_*3*_, the change in porosity was determined, by measuring the combined pore volume displacement of both upstream and downstream pumps as *σ*_*3*_ increased or decreased stepwise^2^.

Three types of experiments were performed, with conditions listed in Table 1:

i) *σ*_*3*_, *σ*_*1*_ and* p*_*p*_ were increased stepwise at a rate of 3 MPa/min while *T* was increased stepwise at 0.1 °C/min, with permeability measurement once all variables reached and stabilized at the setpoint (axis perpendicular to the bedding, sample BS8);
ii) *σ*_*3*_, *σ*_*1*_ and* p*_*p*_ were increased and later decreased stepwise at a rate of 3 MPa/min while *T* was increased and later decreased stepwise at 0.1 °C/min, with permeability measurement once all variables reached and stabilized at the setpoint (axis parallel to the bedding, sample BS13);
iii) *σ*_*3*_ experienced 1.5 cycles of stepwise increase and decrease, followed by 1.5 cycles of stepwise increase and decrease in *p*_*p*_ and finally 1 cycle of stepwise increase and decrease in *T*, with permeability measurement once the changing variable reached and stabilized at the setpoint (axis parallel to the bedding, sample BS16).

In the first two experiments, all variables were changed quasi-simultaneously, representing a full simulation of rock burial (and exhumation) in the Earth’s crust. While axial strain also increases as a result of differential stress, this would only reach a value (< 0.3%) for which we do not expect any effect on permeability (see Fig. 2a in^19^).

In sample BS16 only one variable was changed at the time (while all others were kept constant) to study the effect of that specific variable on permeability and porosity. This experimental sequence was designed to understand how different variables individually and collectively affect the permeability of Bentheim sandstone at different depths under representative pressure/temperature conditions.

## Results

### Rock burial simulation

**Figure 3:**
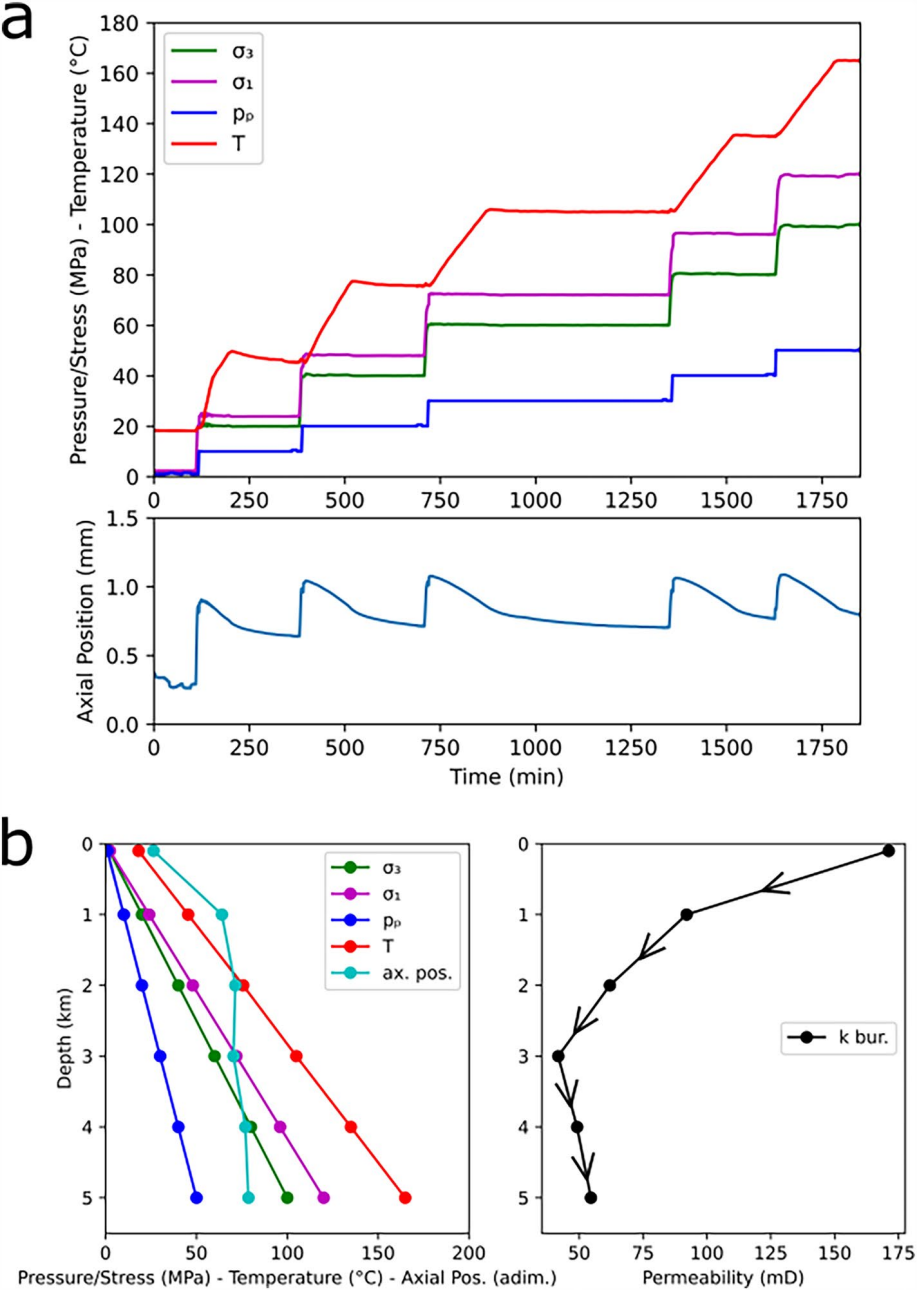
(**a**) time evolution of *σ*_*1*_, *σ*_*3*_, *p*_*p*_ and *T* (top) and axial position (bottom, adimensional for graphic purpose) during the experiment on sample BS8; b) values of *σ*_*1*_, *σ*_*3*_, *p*_*p*_ and *T*, together with the stable axial position (left) and the permeability during a simulated burial at the investigated depths (right).

The effect of rock burial (i.e. increasing depth) on the permeability of BS was investigated on sample BS8, cored with the axis perpendicular to the bedding layers. As the sample was brought to a certain depth, *σ*_*1*_, *σ*_*3*_, *p*_*p*_ and *T* were increased simultaneously, with *T* requiring a longer time to stabilize (Fig. 3a top). The mechanical response of the sample was monitored via the axial position of the piston providing the vertical load and once this was stable (Fig. 3a bottom, increasing values = sample contraction), permeability measurements were performed. Since the internal axial and radial LVDTs proved to be temperature-sensitive, we chose to plot the axial position instead of the axial strain, which was not affected by high temperature, for consistency. The applied conditions are shown in Fig. 3b (left), corresponding to depths of 0.1, 1, 2, 3, 4 and 5 km. These variables correspond to a hydrostatic *p*_*p*_ gradient, a lithostatic *σ*_*3*_ and *σ*_*3*_/*σ*_*1*_ = 0.83 (simulating a reverse-fault regime), and a *T* gradient of 30°C/km. The stable axial position is added to Fig. 3b left, which shows an initial marked contraction followed by alternating (minor) phases of contraction and dilation.

A rapid reduction in permeability is observed at a depth of 1 km, followed by a quasi-linear, slower decrease until depth = 3 km. Here the minimum is observed, with a decrease of 76% compared to the value at a shallow depth. At greater depths, permeability shows a steady smooth recovery, with an increase of 31% at depth = 5 km (Fig. 3b right). Permeability values are shown in Supplementary Table S1.

### Rock burial and exhumation simulations

**Figure 4:**
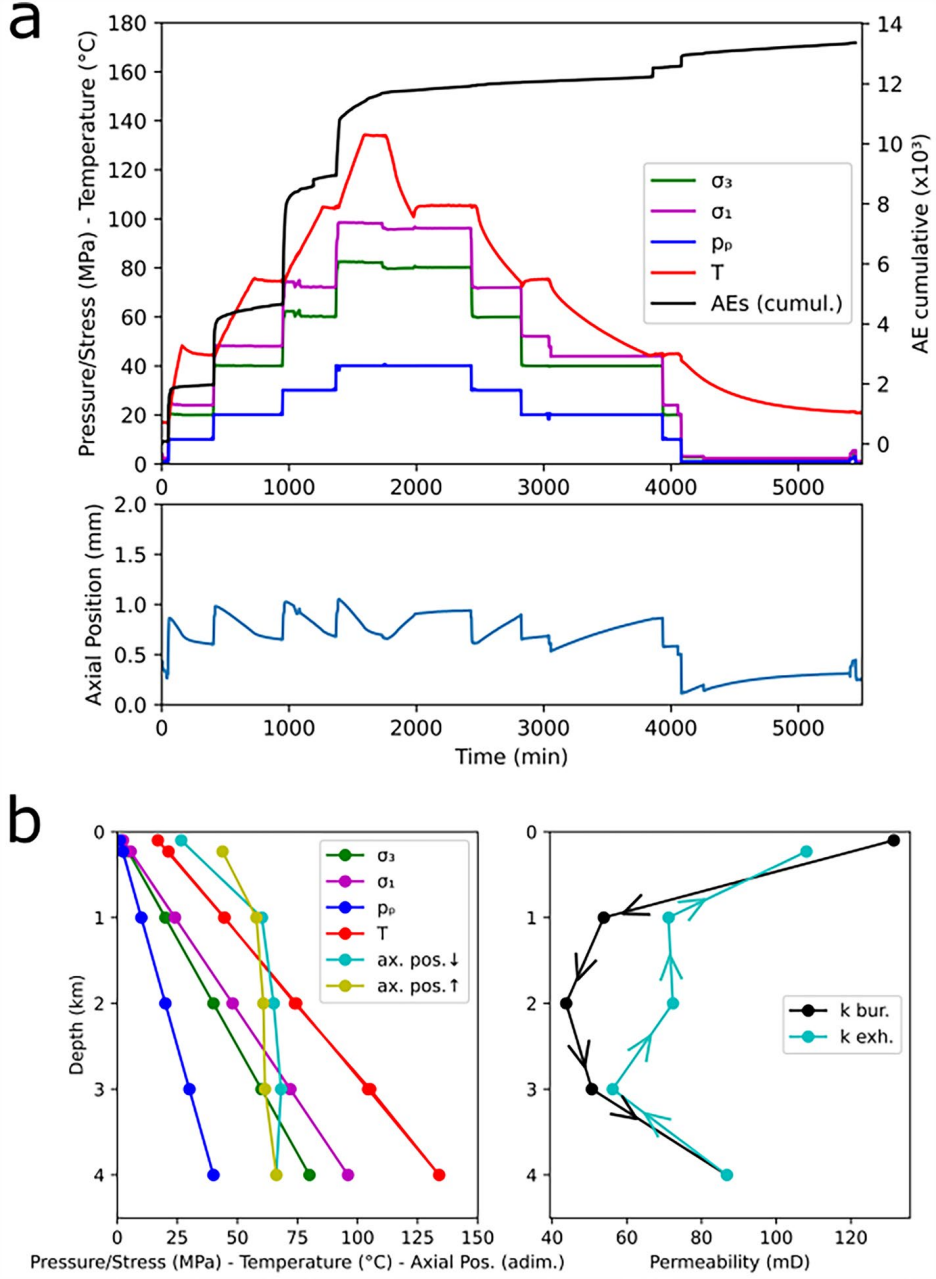
(**a**) time evolution of *σ*_*1*_, *σ*_*3*_, *p*_*p*_, *T* and cumulative AE (top) and axial position (bottom) during the experiment on sample BS13; (**b**) values of *σ*_*1*_, *σ*_*3*_, *p*_*p*_ and *T*, together with the stable axial position (left) and the permeability during simulated burial and exhumation at the investigated depths (right).

Since it has been found that BS was buried down to approx. 3.5 km and later exhumed to shallower depths, the effects of burial and exhumation (i.e. increasing and decreasing depths) were investigated on sample BS13, cored with the axis parallel to bedding. Similar to BS8, in BS13 all variables were simultaneously increased, but after reaching their highest values, they were all reduced step-wise (Fig. 4a top). From this experiment onwards, AE monitoring was added in addition to recording the axial position (Fig. 4a bottom). The investigated depths were 0.1, 1, 2, 3, 4 km during burial and 3, 2, 1 and 0.23 km during exhumation (Fig. 4b left).

The majority of AE activity (shown in Fig. 4a top as cumulative AE) was observed during the stress/pressure step increases. In particular, the highest AE peak is observed when *σ*_*1*_, *σ*_*3*_ and *p*_*p*_ are increased to simulate a depth of 2 km (but the temperature is not yet at the setpoint) and then decreases as deeper conditions are simulated. At the early stage of *T* increases, only negligible AE activity is recorded, while above 80°C (ca. 1200 min) significant AE is recorded also during *T* increases. As unloading/cooling starts, no AE is recorded until *T* ≈ 40°C and when *σ*_*1*_, *σ*_*3*_ and *p*_*p*_ are brought from a simulated depth of 1 km to 0.23 km. As with BS8, also here the sample shows marked axial contraction at shallow depth, while only minor contraction follows at deeper conditions. As exhumation is simulated, the sample shows dilation with values of axial position similar to those reached at the same depth during the burial path (Fig. 4b left).

In terms of permeability (Fig. 4b right), values of this have a quasi-specular behavior to those observed in BS8, although the samples were cut with a different orientation. This confirms that anisotropy has no impact on the permeability evolution of BS^2^. In detail, permeability is reduced largely at shallow depth, reaching a minimum at depth = 2 km (a decrease of 67%), while it partially recovers at depth = 4 km (an increase of 98% compared to the minimum). During the exhumation path, permeability shows a similar but more complex behavior, particularly at depth = 2 km, returning to high values at depth = 0.23 km (only 18% lower than the initial permeability at depth = 0.1 km). Permeability values are shown in Supplementary Table S2.

### Sequential step-wise increase and decrease of ***σ***_***3***_, ***p***_***p***_ and ***T***

To investigate the individual role of each variable, as well as any hysteretic effects on the evolution of permeability with depth, *σ*_*3*_, *p*_*p*_ and *T* were sequential, step-wise increased and decreased in sample BS16. During each step, permeability measurements were conducted. In addition, porosity changes were measured for each *σ*_*3*_ loading and unloading step.

**Figure 5:**
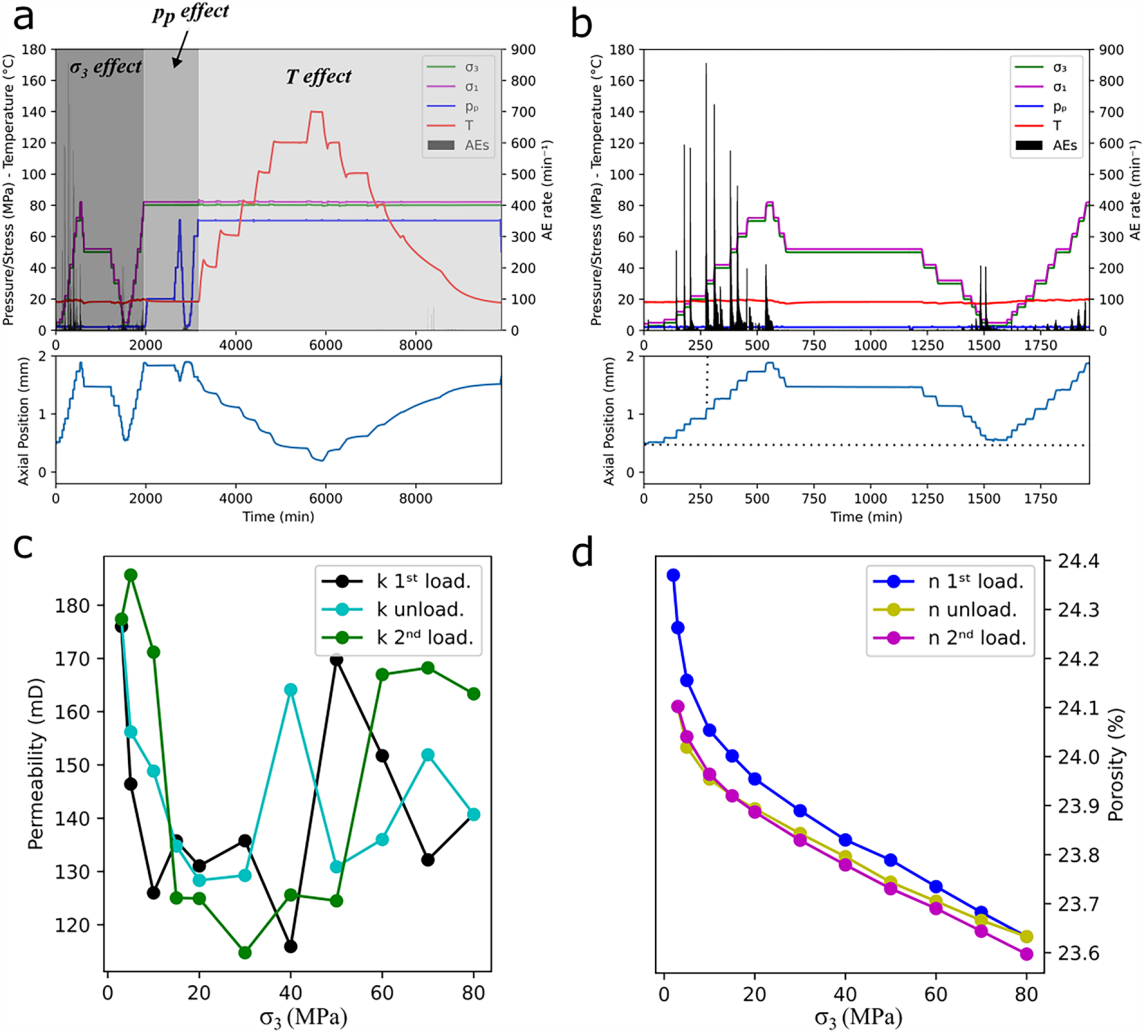
(**a**) time evolution of *σ*_*1*_, *σ*_*3*_, *p*_*p*_, *T* and AE rate (top) and of axial position (bottom) during the experiment on sample BS16; (**b**) snapshot of (**a**) where only the effect of *σ*_*3*_ is investigated; (**c**) values of permeability and (**d**) of porosity at the investigated *σ*_*3*_.

Therefore, the experiment was divided into 3 stages (Fig. 5a). Initially only *σ*_*3*_ was increased and subsequently decreased between 3 and 80 MPa (dark grey shadowed area), while all other variables were kept constant (*σ*_*diff*_ = 2 MPa, *p*_*p*_ = 2 MPa and *T* = 18°C). Once the maximum *σ*_*3*_ was reached during the 2^nd^ loading phase, *p*_*p*_ was increased and subsequently decreased between 2 and 70 MPa (mid-grey shadowed area), again keeping the other variables constant (*σ*_*diff*_ = 2 MPa, *σ*_*3*_ = 80 MPa and *T* = 18°C). Finally, only T was increased from 18 to 140°C (light grey shadowed area, with constant *σ*_*diff*_ = 2 MPa, *σ*_*3*_ = 80 MPa and *p*_*p*_ = 70 MPa).

During the first stage, the vast majority of AE activity occurred during the initial loading, with a peak of over 800 AEs/min recorded when *σ*_*3*_ was raised to 30 MPa. After this peak, AE activity declined, reaching a rate of approx. 200 AEs/min during the final step. During unloading, AE activity was absent and resumed only when *σ*_*3*_ was lowered to the last 3 steps (i.e. 10, 5 and 3 MPa) with rates of 200 AEs/min. During the second loading phase, an ever-increasing, but with lower rates, AE activity is observed reaching a peak of 100 AEs/min at the last step (i.e. 80 MPa, Fig. 5b top). In terms of deformation, in Fig. 5b (bottom) the contraction and dilation phases can be observed during loading and unloading respectively. Most of the contraction occurred within *σ*_*3*_ < 30 MPa (axial position = 1.09 mm, see the vertical dotted line, compared to 1.88 mm reached at *σ*_*3*_ = 80 MPa). In addition, after the unloading phase, the sample showed irreversible deformation, since the axial position at the end of this phase is higher than that at the beginning of the loading phase (0.54 mm vs 0.51 mm, see horizontal dotted line).

During the 1^st^ loading phase, permeability shows a 34% decrease from its initial value as *σ*_*3*_ rises to 40 MPa, while values at higher *σ*_*3*_ follow an oscillating trend with the value at *σ*_*3*_ = 80 MPa being 20% than the starting value. During the unloading phase, permeability has again an oscillatory trend until *σ*_*3*_ = 20 MPa, before monotonically increasing towards approximately its initial value during the first loading phase. Permeability behaves similarly during the 2^nd^ loading phase, reaching a minimum at *σ*_*3*_ = 30 MPa. Once again *k* shows an oscillatory pattern at higher *σ*_*3*_ (Fig. 5c). In terms of porosity, during all phases n decreases and increases linearly for *σ*_*3*_ > 20 MPa, reaching at the end of the 1^st^ loading phase a value of 23.63% (a reduction of 3% to the initial value). At the end of the unloading and the 2^nd^ loading phases, one can notice that their respective final values are lower than those reached in the previous phases (0.67 and 0.15 %), suggesting a loss of pore space with each loading/unloading phase (Fig. 5d).

**Figure 6:**
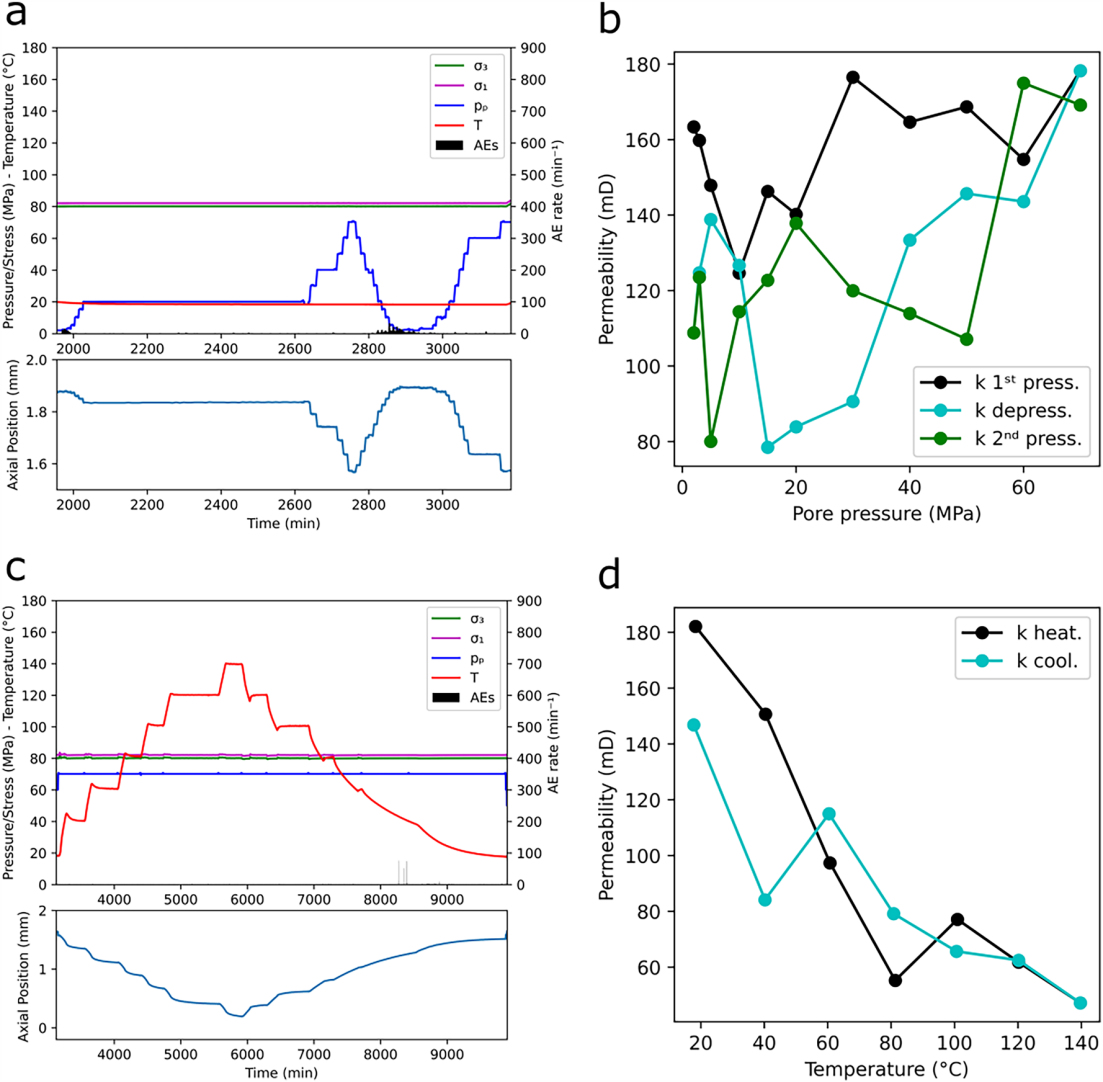
(**a**) snapshot of Fig. 5a, showing time evolution of *σ*_*1*_, *σ*_*3*_, *p*_*p*_, *T* and AE rate (top) and of axial position (bottom) during the experiment on sample BS16, where only the effect of *p*_*p*_ is investigated; (**b**) values of permeability at the investigated *p*_*p*_; (**c**) snapshot of Fig. 6a, where only the effect of *T* is investigated; (**b**) values of permeability at the investigated *T.*

During the second stage (i.e. pore space pressurization and depressurization) low AE activity with peaks < 20 AEs/min was recorded only at *p*_*p*_ < 20 MPa (corresponding to *σ*_*3*_ > 60 MPa) during the 1^st^ pressurization and the depressurization. In contrast, no AEs were recorded during the 2^nd^ depressurization (Fig. 6a top). In terms of deformation, sample dilation and contraction were associated with pressurization and depressurization phases respectively. The values of the axial position reached in a phase equaled those reached in the previous phase at the same level of *p*_*p*_ (Fig. 6a bottom), suggesting reversible deformation of the sample with changing *p*_*p*_. The behavior of the permeability during this stage is complex, but it can be split into two parts for both pressurization and depressurization phases: i) at low *p*_*p*_ (5–15 MPa) permeability decreases, reaching the minimum value (*k* decreases of 23–26%); ii) at higher *p*_*p*_, permeability increases reaching a value higher than that at *p*_*p*_ = 2 MPa (starting value) and 43–112% higher than the minimum *k* reached in each phase (Fig. 6b).

During the third stage (i.e. heating and cooling), AE events only appeared during the cooling phase when *T* reached 40°C. Three AE rate peaks were recorded, having a rate of 78, 56 and 72 AEs/min respectively (Fig. 6c top). The sample dilated during cooling and contracted during heating, with no difference in axial position between the beginning of the heating and the end of the cooling phase (i.e. reversible deformation, Fig. 6c bottom). Overall, permeability shows an inverse relationship with *T*, both during the heating and cooling phases, with a 74% reduction from the beginning to the end of the heating. However, the decline in permeability is non-linear and can be divided into two segments: i) fast, monotonic decrease up to *T* = 80°C (70% reduction) and ii) slow, oscillating decrease for *T* > 80°C (15% reduction). During the cooling, a marked increase in *k* occurred between values at *T* = 40°C and *T* = 18°C (74 %), when the AE peaks were recorded, with the post-cooling permeability being 19% lower than the pre-heating permeability (Fig. 6d).

All permeability values are shown in Supplementary Table S3.

## Discussion and conclusions

The goal of the characterization of a georeservoir rock is to understand the properties and behaviors of such rock at conditions representative of the depth at which the rock lies. Studying the effect of the effective pressure (*σ*_*eff*_) on the permeability (*k*), as has been conducted previously on Bentheim Sandstone (BS), is a necessary but not sufficient requirement for a rock found at depths of 2 km and previously buried down to 3.5 km. Therefore, in this study pore pressure (*p*_*p*_), temperature (*T*) and history effect have been also investigated.

Although remaining high (tens to hundreds of mD), the permeability of BS shows a clear correlation with depth, reaching a minimum at depths of 2–3 km and slowly increasing at greater depths. When an exhumation path is simulated, permeability shows a more complex pattern but is still characterized by a general decrease from 4 to 2 km deep, followed by an overall increase toward shallower conditions. To understand the processes controlling such behavior, it is fundamental to study individually each investigated variable and how they contribute to the evolution of permeability with depth.

The first investigated variable was the horizontal stress (*σ*_*3*_), while previous studies considered *σ*_*eff*_, which depends on *σ*_*3*_ since in any case *p*_*p*_ is kept constant. The goal here is to show the effect of a changing independent variable, which in the first stage is *σ*_*3*_ and in the second is *p*_*p*_. In both cases *σ*_*eff*_ changed, but a result of one changing and one constant independent variable. Therefore, we can compare our results in terms of *σ*_*3*_ with previous results in terms of *σ*_*eff*_. Similar to Dautriat et al.^16^, we observed an effect of *σ*_*3*_ on the BS permeability, particularly at low-stress levels (< 30 MPa), where most of the sample contraction occurs. This agrees with the higher amount of axial deformation, an ever-increasing amount of AE activity and the fast, non-linear reduction in porosity in the region 3 < *σ*_*3*_ < 30 MPa (34%). Similar behavior has been also observed on smaller samples (100 × 40 mm) of Crab Orchard Sandstone (COS), a less porous and permeable rock. In fact, for samples cored parallel to the bedding layers, permeability decreases quasi-linearly (67%) with *σ*_*eff*_ < 40 MPa, while for higher stresses permeability seems unaffected by *σ*_*eff*_^23^. Dautriat et al.^16^ argued that the initial non-linear decrease in porosity and the apparent inverse relationship between permeability and *σ*_*eff*_ is caused by end effects at the interface piston/sample. These will also explain the irreversible deformation of BS at the end of the following unloading and loading phase. While agreeing with this interpretation, even so, our samples are three times longer, we believe that the microstructure plays a role too, to justify the similar pattern but different amount of permeability reduction with *σ*_*3*_ between BS and COS. COS is an anisotropic rock with visible bedding layers and a high aspect ratio (microcrack) pore space, which enhances pore space closure and therefore permeability. On the other end, BS is an isotropic rock, with invisible (to the naked eye) bedding layers and a low aspect ratio (equant pore) pore space, which impedes pore space closure. Since both COS and BS samples were cored parallel to the bedding layers, and therefore perpendicular to the piston/sample interface, we cannot justify the permeability reduction solely with the end effects. In addition, we also observed that permeability recovers at the end of the unloading phase and the pore space is reduced by 0.67 and 0.15% after the unloading and 2^nd^ loading phase respectively. We conclude that the permeability reduction at low-stress levels is a combination of end effects at the piston/sample interface and the microcrack closure (as per^28^). However, with the current experimental apparatus, we cannot quantify the contribution of these two factors. At higher stresses, the equant pore space of BS impedes significant sample contraction and so the permeability is mostly unaffected by *σ*_*3*_. Therefore, although marginal, permeability shows an inverse relationship with *σ*_*3*_ at low-stress levels.

The second investigated variable was the pore pressure (*p*_*p*_). According to Guéguen and Palciauskas^14^, we expected permeability and *p*_*p*_ to be directly proportional. This relationship has not previously been studied on BS, and our results do not provide a straightforward answer. According to the deformation data, the sample BS16 (Fig. 6a) dilated with increasing *p*_*p*_, although not reaching the same level of axial position at the same *σ*_*eff*_ observed while *σ*_*3*_ was increasing (*σ*_*eff*_ = 10–2 = 8 MPa, with *σ*_*3*_ = 10 MPa vs *σ*_*eff*_ = 80–70 = 10 MPa, with *p*_*p*_ = 70 MPa). At pore pressure values from approximately 5–15 MPa, we observe a complex, overall increase in permeability. However, at low *p*_*p*_, an inverse relationship between *k* and *p*_*p*_ was observed. The explanation for this may come from the presence of clay minerals. Coyner^22^ found that clay minerals in the rock matrix may be dislodged, clogging the pore space when *p*_*p*_ is increased from 0 to 16 MPa, therefore reducing the permeability. Coyner^22^ did not test whether this effect persisted at higher pressures. Tchistiakov^20^ confirmed, through SEM images, the release of clay particles and their successive deposition in narrower pore throats, which lowered the permeability of BS at different flow rates. We believe that these findings, in combination with our results, may explain the complex behavior of the *k*–*p*_*p*_ relationship: an initial clay-clogged-dominated pore space at low *p*_*p*_ and consequent reduction in *k*, is followed by a dilated-dominated pore space at higher *p*_*p*_ and consequent increase in *k*. Successive cycles of depressurization and pressurization have a similar pattern, but with minima at different *p*_*p*_ levels and a general more oscillating behavior at low *p*_*p*_, probably still caused by the presence of dispersed particles of clay minerals.

The third investigated variable was the temperature (*T*). Here the relationship seems clearer: permeability decreases with increasing *T* and increases with decreasing *T*. In fact, the absence of AE activity during the heating phase indicates the absence of microcracking processes, suggesting instead a thermal expansion of the mineral matrix. This behavior agrees with the high porosity nature of BS. During the cooling phase, the *k*-*T* curve follows a similar path, but when thermal microcracking occurs at *T* < 40 °C, the permeability increases significantly. We conclude that an inverse-proportionality, caused by the thermal expansion of the mineral matrix exists between *k* and *T*. When present, thermal microcracking can boost the permeability.

**Figure 7:**
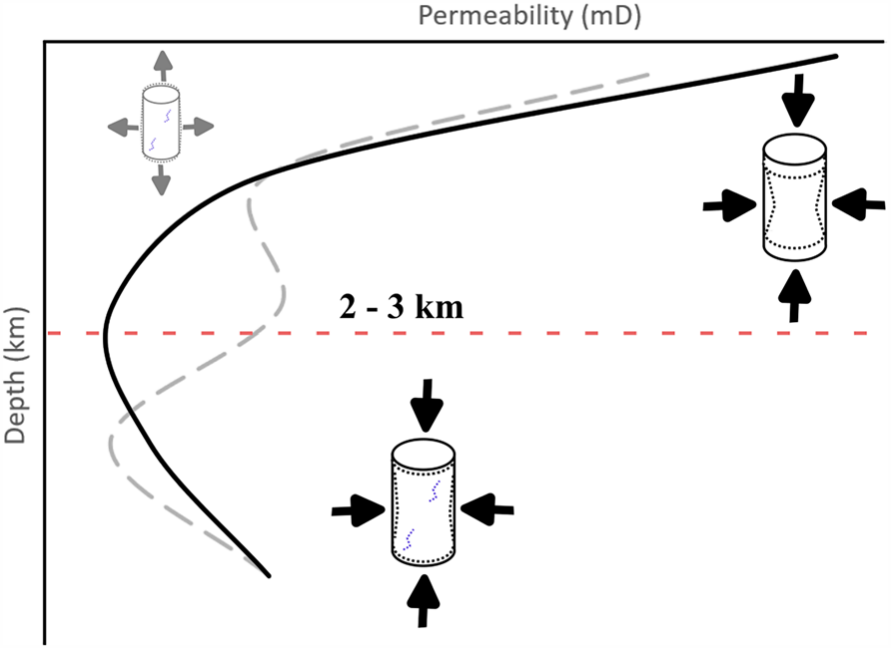
sketch showing the evolution of BS permeability with depth, during the simulated burial (solid black line) and exhumation (dashed grey line) paths. The minimum value in permeability during the burial path, around depths of 2–3 km, is marked by the dashed red line.

Finally, with these findings, we can understand the behavior of BS permeability with increasing/decreasing depth, as shown in Fig. 7. During the initial burial path, the sample experiences: i) high contraction caused by increasing *σ*_*3*_, only partially attenuated by *p*_*p*_, ii) dispersion of clay minerals and redeposition caused by increasing *p*_*p*_, and iii) thermal expansion of the mineral matrix caused by increasing *T*. All of these processes have a negative effect on the permeability, which decreases by 50–70% reaching a minimum at conditions representative of depths of 2–3 km. Beyond this point, the sample experiences: i) low contraction caused by the further increase of *σ*_*3*_ and *p*_*p*_, and ii) microcracking instead of thermal expansion of the mineral matrix (as shown by the occurrence of AE during the heating in Fig. 4a) caused by the further increase of *T*. The consequence of these is an increase of permeability to intermediate values in the range of the simulated depths. During the initial exhumation path, no significant recovery in permeability occurs, since irreversible damage and thermal contraction balances out the dilation caused by the reduction of the vertical and lithostatic stress. However, at shallower conditions, as *T* further decreases, a cooling-dominated microcrack occurs. This will cause a sudden increase in permeability to values closer to the pre-burial ones.

The results and discussion presented in this study show how important is not only to understand the effect of one individual variable at the time on the permeability but also how the interaction of different variables together and their past history affect the permeability of Bentheim Sandstone. In particular, while remaining at high values of permeability, at the maximum depths at which BS currently lies, permeability is more the half of that observed at the surface. This has clear implications when assessing the potential of BS as a georeservoir, since its permeability could be overestimated at certain depths. While the permeability of BS does not change significantly, the permeability of other rocks does. In conclusion, our findings have likely greater importance to other georeservoir rocks, whose permeability may be more affected by the interaction of different variables, and so to decrease the risk of incorrect permeability estimations.

### Supplementary Information

Supplementary Information.

## Data Availability

The datasets used and/or analyzed during the current study are available from the corresponding author upon reasonable request.
