# Supplementary material for: Permeability evolution of Bentheim Sandstone at simulated georeservoir conditions
Source: Sci Rep. 2023 Sep 27;13:16171. doi: 10.1038/s41598-023-42826-3 (PMC10533886; doi:10.1038/s41598-023-42826-3)
Supplement: Supplementary file 1 — Supplementary Information. [file 41598_2023_42826_MOESM1_ESM.pdf]

Supplementary Information

Article in *Scientific Reports*

Permeability evolution of Bentheim Sandstone at simulated georeservoir conditions

[1]Marco Fazio, [2]Michael R. Chandler and [1,3]Martin Sauter

[1]*Department of Applied Geology, University of Göttingen, Göttingen, Germany*

[2]*School of Geosciences, University of Edinburgh, Edinburgh, UK*

[3]*Leibniz-Institute of Applied Geophysics, Hannover, Germany*

Corresponding author: M. Fazio, Department of Applied Geology, University of Göttingen, Goldschmidtstrasse 3, Göttingen, 37077, Germany. (marco.fazio@uni-goettingen.de)

Contents of this file

Table S1, S2, S3

Introduction

The supplementary material consists of:

- Table S1, showing the permeability values measured during the experiment on sample BS8;
- Table S2, showing the permeability values measured during the experiment on sample BS13;
- Table S3, showing the permeability values measured during the experiment on sample BS16.

Supplementary Table S1: experimental conditions and permeability values on test BS8

**Sim. depth (km)**	**σ_3 (MPa)**	**σ_1 (MPa)**	**p_p (MPa)**	**T (°C)**	**k (mD)**
0.1	2	2.4	1	18.18	171.12
1	20	24	10	45.40	92.03
2	40	48	20	75.70	62
3	60	72	30	104.98	41.78
4	80	96	40	134.99	49.13
5	100	120	50	164.85	54.58

Supplementary Table S2: experimental conditions and permeability values on test BS13

**Sim. depth (km)**	**σ_3 (MPa)**	**σ_1 (MPa)**	**p_p (MPa)**	**T (°C)**	**k (mD)**
0.1	2	2.4	1	16.85	131.45
1	20	24	10	44.44	53.82
2	40	48	20	74.48	43.75
3	60	72	30	104.39	50.64
4	80	96	40	133.95	86.77
3	60	72	30	105.27	56.22
2	40	48	20	73.92	72.33
1	20	24	10	44.65	71.16
0.23	4.6	5.5	2.3	21.27	108.08

Supplementary Table S3: experimental conditions and permeability values on test BS16

1st stage		***2nd stage***		***3rd stage***	
**σ_3 (MPa)**	**k (mD)**	**p_p (MPa)**	**k (mD)**	**T (°C)**	**k (mD)**
3	176.01	2	163.36	18.33	182.08
5	146.42	3	159.76	40.38	150.67
10	126.00	5	147.86	60.73	97.37
15	135.76	10	124.65	81.3	55.21
20	131.04	15	146.27	100.92	77.11
30	135.78	20	140.21	120.23	61.76
40	115.91	30	176.50	139.7	47.18
50	169.79	40	164.63	120.23	62.47
60	151.74	50	168.67	100.62	65.66
70	132.18	60	154.74	80.78	79.17
80	140.72	70	178.21	60.43	114.97
70	151.91	60	143.56	40.23	84.15
60	136.02	50	145.69	17.74	146.83
50	130.86	40	133.37		
40	164.14	30	90.59		
30	129.27	20	83.87		
20	128.33	15	78.47		
15	134.77	10	126.67		
10	148.85	5	138.81		
5	156.20	3	124.72		
3	177.39	2	108.80		
5	185.66	3	123.48		
10	171.19	5	80.01		
15	125.04	10	114.39		
20	124.93	15	122.72		
30	114.76	20	137.81		
40	125.61	30	119.95		
50	124.50	40	113.92		
60	166.97	50	107.11		
70	168.23	60	174.97		
80	163.36	70	169.15		
